# Usability of the 4Ms Worksheet in the Emergency Department for Older Patients: A Qualitative Study

**DOI:** 10.5811/westjem.18088

**Published:** 2024-01-25

**Authors:** Mackenzie A. McKnight, Melissa K. Sheber, Daniel J. Liebzeit, Aaron T. Seaman, Erica K. Husser, Harleah G. Buck, Heather S. Reisinger, Sangil Lee

**Affiliations:** * University of Iowa, Roy J. and Lucille A. Carver College of Medicine, Iowa City, Iowa; † University of Iowa, College of Nursing, Iowa City, Iowa; ‡ University of Iowa, Roy J. and Lucille A. Carver College of Medicine, Department of Internal Medicine, Iowa City, Iowa; § Penn State University, Ross and Carol Nese College of Nursing, University Park, Pennsylvania; ∥ Department of Epidemiology, University of Iowa College of Public Health, Department of Emergency Medicine, Iowa City, Iowa

## Abstract

**Introduction:**

Older adults often have multiple comorbidities; therefore, they are at high risk for adverse events after discharge. The 4Ms framework—what matters, medications, mentation, mobility—has been used in acute and ambulatory care settings to identify risk factors for adverse events in older adults, although it has not been used in the emergency department (ED). We aimed to determine whether 1) use of the 4Ms worksheet would help emergency clinicians understand older adult patients’ goals of care and 2) use of the worksheet was feasible in the ED.

**Methods:**

We conducted a qualitative, descriptive study among patients aged ≥60 years and emergency clinicians from January–June 2022. Patients were asked to fill out a 4Ms worksheet; following this, semi-structured interviews were conducted with patients and clinicians separately. We analysed data to create codes, which were divided into categories and sub-categories.

**Results:**

A total of 20 older patients and 19 emergency clinicians were interviewed. We identified two categories based on our aims: understanding patient goals of care (sub-categories: clinician/ patient concordance; understanding underlying goals of care; underlying goals of care discrepancy) and use of 4Ms Worksheet (sub-categories: worksheet to discussion discrepancy; challenges using worksheet; challenge completing worksheet before discharge). Rates of concordance between patient and clinician on main concern/goal of care and underlying goals of care were 82.4% and 15.4%, respectively.

**Conclusion:**

We found that most patients and emergency clinicians agreed on the main goal of care, although clinicians often failed to elicit patients’ underlying goal(s) of care. Additionally, many patients preferred to have the interviewer fill out the worksheet for them. There was often discrepancy between what was written and what was discussed with the interviewer. More research is needed to determine the best way to integrate the 4Ms framework within emergency care.

Population Health Research Capsule What do we already know about this issue?

*Older adults with multiple comorbidities face high risks post-discharge. The 4Ms framework—what matters, medication, mentation, and mobility—is used in various settings.*

What was the research question?

*Can the use of the 4Ms framework in the ED setting help clinicians understand older adult patients’ goals of care, and is it feasible?*

What was the major finding of the study?

*Rates of concordance between patient and clinician on the main concern/goal of care and underlying goals of care were 82.4% and 15.4%, respectively.*

How does this improve population health?

*Integration of the 4Ms framework in emergency care could enhance understanding and alignment with older adults’ underlying goals of care.*

## INTRODUCTION

Older adults will account for over 20% of the US population in the next decade, and they are more likely to have multiple comorbidities, take more medications, and use more healthcare resources than individuals in younger age groups.^1^ The visit rate to emergency departments (ED) in the US in 2019 totaled 43 visits per 100 for individuals 65–74 years of age and 66 visits per 100 for individuals ≥75 years. The visit rate for those ≥75 was higher than all other age groups (ranging from 19–25 visits per 100 individuals) except for those under one year of age. Several studies have shown that there is a lack of recognition of risk factors (eg, polypharmacy, fall risk, delirium) for adverse outcomes among older adults in the ED.^2^ ^–^ ^4^ Several assessment tools have been developed in the ED to evaluate risk factors (eg, identification of seniors at risk^5^), but there is no tool to effectively communicate the needs of older adults.^6^ ^–^ ^8^

The 4Ms framework of age-friendly health systems was created by the John A. Hartford Foundation in collaboration with the Institute for Healthcare Improvement, American Hospital Association, and Catholic Health Association. It incorporates four key elements: *what matters, medication, mentation*, and *mobility*.^9^ These four elements were developed with current evidence-based practices with the intention of guiding clinician conversations with older adults. The 4Ms framework considers the common risk factors for adverse outcomes in older adult patients (eg, risk for delirium, potentially inappropriate medications, and challenges with mobility).

While there has been a focus on implementing the 4Ms framework in acute and ambulatory care settings, there have been few studies on its potential application in the ED.^10^ The ED is one clinical setting in which it could be important to discuss the 4Ms so that all potential risk factors are identified and care is tailored to the needs of older patients. There is often a time constraint for the emergency clinician to engage in a lengthy conversation about what matters to the patient. (“What matters” entails discussing the specific details that matter to each patient on a deep level, including their goals and preferences for care, which can guide the care team and align care to what truly matters to the patient.) Use of the 4Ms framework could potentially prevent adverse outcomes for older adult patients receiving care in the ED by recognizing risk factors such as polypharmacy, fall risk, and delirium.

The 4Ms worksheet was developed by the team at Age-Friendly Care, PA, a Geriatric Workforce Enhancement Program at the Penn State Ross and Carole Nese College of Nursing. The worksheet is a patient-facing tool that allows individuals to identify what matters to them and what questions they may have about potential problems with mobility, mentation, and medications. The tool was developed to help facilitate conversations between patients and clinicians, but its use has not yet been evaluated in the ED (Appendix 1). We aimed to evaluate the potential usability of the 4Ms worksheet in the ED to facilitate conversations about what matters, medications, mention, and mobility between older patients and emergency clinicians, and to assess whether the 4Ms worksheet may support emergency clinicians’ understanding of patients’ goals of care, including barriers and facilitators to using the worksheet.

## METHODS

### Study Design

We conducted the study using a qualitative, descriptive approach involving semi-structured interviews with older adult patients (aged ≥60 years) and their emergency clinician from January–June 2022. This study is part of a larger study that examined patient goals reported through the 4Ms worksheet using the qualitative method.^11^ ^–^ ^13^ In the present analysis we focused on usability of the worksheet by patients, as well as emergency clinicians’ understanding of patients’ goals of care.^11^ The local institutional review board approved this study and determined it to be exempt. We adhered to the Consolidated Checklist for Reporting Qualitative Research (COREQ) guidelines. (Appendix 2).

### Study Setting

This study took place in a single academic ED with Level I trauma accreditation and an annual census of approximately 60,000 patients. Clinicians practicing in this specific ED include attending physicians, residents, and advanced practice providers (APP). The ED has a three-year emergency medicine residency program and an 18-month fellowship for APPs; any EM resident or APP whom the patients saw would be enrolled in of one of these training programs. The institution is currently a part of the age-friendly health systems movement.

### 4Ms Worksheet

The 4Ms worksheet was created by Age-Friendly Care, PA at the Penn State Ross and Carol Nese College of Nursing, which is a Center of Geriatric Nursing Excellence. The worksheet explains each category of the 4Ms framework—what matters, medications, mentation, and mobility— a program housed in the age-friendly healthcare system and provides space for the patient to write a short answer response about each.

### Participants and Sampling

Participants were recruited for an interview if 1) they were aged ≥60 years; 2) they were currently receiving care in the ED; 3) their chief complaint was not related to altered mental status; 4) they were able to read and understand the 4Ms worksheet written in English, and 5) they had already been evaluated by a clinician in the ED. Patients who met eligibility criteria were approached by a member of the research team to provide study information. Consent was obtained from all participants before beginning the interview.

### Data Collection

Two medical students, MM and MS—who were trained by the primary investigator SL and by DL who has extensive experience with qualitative research—conducted semi-structured interviews with patients and clinicians. The research team (SL, MS) developed an interview guide (Appendix 3). Interview responses were captured as handwritten notes instead of by digital recording due to cost. The research team collected patient age, gender, and type of clinician interviewed (staff physician, resident physician, or APP). Interviewers also took field notes, which contained the reason for the visit and contextual factors (symptom relief, diagnostic test, disposition, non-verbal aspects of the interview). The interviewer entered all data into REDCap, a secure electronic data capture tools hosted at The University of Iowa Hospitals and Clinics.^14^ No compensation was provided for interviews.

Patient interviews took place in the room where the patient’s ED evaluation took place. The emergency clinician was not present for the patient interview. First, the patient completed the 4Ms worksheet either independently or verbally to a member of the research team, followed by discussion. (Patients were not asked whether they had completed a 4Ms worksheet in prior healthcare encounters.) Discussion included patient goals for the current ED visit, questions about each category within the 4Ms framework, and how the patient felt their visit had gone overall. At the end of the interview, the interviewer offered the patient to keep the 4Ms worksheet for use as a reference in future healthcare encounters. Patient interviews lasted 30–60 minutes (including the time spent completing the 4Ms worksheet). After the patient interview was complete, the patient’s clinician was interviewed about their perception of the patient’s goals of care and how those goals were elicited during patient assessment. Clinician interviews occurred without the patient present and lasted 1–5 minutes.

### Analysis

Interview data stored in REDCap was analyzed by research team members SL, MM, MS, and DL using content analysis.^15^ Each interview was initially coded by two members (MS, MM), who received a brief training on qualitative content analysis. Two faculty investigators (SL and DL) reviewed these codes and made further recommendation before consensus was reached. The entire research team met to discuss coding; any discrepancies were resolved through group discussion. Identified codes were entered into a codebook (Appendix 4), which was maintained and updated throughout data analysis. Codes were grouped into categories and sub-categories to describe the data. Data collection and analysis followed an iterative process and occurred simultaneously, which allowed for revisions to the interview guide to address gaps in the data. Interviews continued until the research team jointly determined that no new information relevant to the research aims was emerging.

### Rigor

Data was analyzed by a research team with a variety of backgrounds to reduce individual bias and improve credibility of the results.^16^ ^,^ ^17^

## RESULTS

We approached 21 patients to conduct semi-structured interviews; one declined due to unknown reasons. In total we interviewed 20 patients and 19 clinicians during the six-month period. It should be noted that the reason for the small sample size in a six-month period was due to interviewers MM and MS also having medical school duties. All but one patient had a clinician to interview; the original clinician for that one patient was no longer in the ED, and the new clinician was unable to answer the questions, as the patient was being discharged. Nine attending physicians, eight residents, and one APP participated in the study (Table 1). The interview process for patient took about 30–60 minutes (median 45 minutes), including time to fill out the 4Ms worksheet, and 1–5 minutes (median 3 minutes) to interview emergency clinicians.

**Table 1.** Patient and emergency clinician demographic data.

Variable	*N*	Percent (%)
Patient age (years)		
60–70	8	40
70–80	9	45
80–90	2	10
90+	1	5
Patient gender		
Male	9	45
Female	11	55
Clinician role		
Attending physician	9	45
Resident	8	40
Advanced practice provider	1	5
Unknown	1	5

Patient and clinician interviews resulted in three themes on the topic of understanding patient goals of care: clinician/patient concordance; understanding underlying goals of care; and underlying goals of care discrepancy (summarized in Table 2). The 4Ms worksheet was used to facilitate conversations about what matters, mentation, mobility, and medications, which support understanding of patients’ goals of care, including barriers to and facilitators of its use (Appendix 4).

**Table 2.** Categories and sub-categories for understanding patient goals of care and utilization of the 4Ms worksheet.

Categories	
Understanding patient goals of care	Utilization of 4Ms worksheet
Clinician/patient concordance	Worksheet to discussion discrepancy
Understanding underlying goals of care	Challenges using the worksheet
Underlying goals of care discrepancy	Challenge completing the worksheet before discharge

### Understanding Patient Goals of Care

#### Clinician/patient concordance

In many cases, patients and clinicians arrived at concordant perceptions of the goals of care. This was indicated when patient and clinician agreed on the main concern and goals of care for the visit, such as in the following examples:

In interview 2, both the patient and clinician agreed that the main concern was addressing the patient’s fever and coordinating care to address recurrent fevers related to chemotherapy; in interview 3, both the patient and clinician agreed that the goal of care was evaluation after fall and being able to continue living independently; in interview 4, both the patient and clinician agreed that the goal of care was addressing symptoms of constipation and abdominal pain; in interview 9, both the patient and clinician agreed that the main concern that brought the patient to the ED was chest pain; in interview 10, both the patient and clinician agreed that the goal of care was addressing symptoms of fatigue; and in interview 11, both the patient and clinician agreed that the goal of care was ruling out serious cardiac pathology. In this last case, both the patient and clinician identified a fear that the patient’s chest pain may have pointed to serious cardiac pathology given the patient’s history. The clinician elicited the foreboding feeling that the patient was having.

In interview 14, both the patient and clinician understood that the patient’s chest pain was what mattered most. In interview 15, both the patient and clinician wanted to address abdominal pain. In interview 16, both the patient and clinician agreed that the main goal of care was pain management. In interview 17, the patient felt that they were treated well and when asked whether the clinician had addressed their concerns, answered, “Yeah, everyone was nice.” However, for this patient, there was no clinician perspective to compare to. In interview 20, the patient reported that she was kept up to date (on her care) and felt that the ED clinician did “just fine” in addressing her goals of care, questions, and concerns.

Overall, 14 of 19 patients and clinicians agreed on the main concern or goals of care for the visit (Table 3). Further, we grouped these into symptom evaluation (Interviews 2, 4, 11); symptom management (Interview 16); symptom evaluation/management (Interview 3); generic agreement (Interviews 9, 10, 14); and miscellaneous (interviews 17, 20).

**Table 3.** Clinician understanding of patient goals of care.

	Attending n = 10, (%) response = yes	Resident (MD/DO or APP) n = 8, (%) response = yes	APP n = 1, % response = yes	Concordance (%)
Patient perspective on whether clinician understood goals of care	4 (40)	5 (62.5)	NA	NA
Was the main concern addressed by clinician?	7 (70)	6 (75)	1 (100)	82.35
Was the underlying goal of care addressed by clinician?	2 (20)	0 (0)	0 (0)	15.38

*APP*, advanced practice provider.

### Understanding Underlying Goals of Care

In some cases, the patient and clinician agreed on underlying goals of care for the patient. An underlying goal of care is defined as aspects of what matters to the patient in their daily life or health that affect their main concern and goal of care. Examples are as follows: In interview 2, both the patient and clinician suggested that a related goal of care was to coordinate with the team managing the patient’s cancer to develop a care plan going forward that would address their recurrent fevers and chemotherapy issues; and in interview 3, both the patient and clinician agreed that an underlying goal of care was to be able to continue living independently. Overall, 2 of 19 patients and clinicians agreed on underlying goals of care (Table 3).

### Underlying Goals of Care Discrepancy

Despite agreeing on main concerns and goals of care, patients and clinicians often did not agree on underlying goals of care. This was the case when interpretation of what matters for the patient and clinician was discrepant, as shown in these examples: In interview 5, the clinician mentioned that the main goal of care was pain relief, and that the patient would rather be at home and “soil himself” than be at the [deidentified] hospital. The patient’s main goal of care was to work on physical therapy, gain strength, and get off some medications. Thus, we identified themes of medication concerns and maintaining independence from this interview. In interview 4, the clinician identified improving symptoms as the main goal of care, but the patient identified the main goal as improving independence. Thus, we identified theme of medication concerns from this interview.

During the 19 clinician interviews, 17 were able to identify the main concern that brought the patient to the ED. Of these 17 clinicians, 14 (82.35%) mentioned a main concern or goal that matched with the patient’s goal. Thirteen clinicians mentioned an underlying goal of care for the patient. Of these 13, only two clinicians (15.38%) mentioned an underlying goal of care that matched with the patient’s underlying goals (Table 3). The responses on patient perspective, main concern, and underlying goals of care were similar between attending and resident physicians.

### Utilization of the 4Ms Worksheet

Implementation of the 4Ms worksheet revealed multiple potential barriers to and facilitators of its use, including worksheet to discussion discrepancy, challenges using the worksheet, and challenge completing the worksheet before discharge (Table 2).

### Worksheet to Discussion Discrepancy

In some cases, patients’ answers to prompts on the 4Ms worksheet did not match information obtained through discussion with the research team, as shown in these examples: In interview 4, the patient answered “no” to medication concerns, but discussed many issues related to medications; in interview 3, the patient discussed information relevant to discharge/disposition and revealed opportunity to learn about medications that was not captured in the worksheet answers; and in interview 20, the patient wrote “no” to medication concerns, but had concerns about two of their medications. These examples could indicate that there was not enough space on the worksheet to provide the information, or that the patient did not care to fill out the worksheet in detail. A possibility also exists that the patient was reminded of more details through discussion that they did not think about before.

### Challenges Using the Worksheet

Many patients preferred a verbal discussion about what mattered to them in their care as opposed to filling out the worksheet. Nine of 20 participants did not feel comfortable with filling out the 4Ms worksheet, and interviewers offered to fill it in for them. Some appeared to be uneasy completing the worksheet, as shown in these examples: Patient 2 began fidgeting with the worksheet and expressed discomfort with filling it out, stating that unease with worksheets extended back to being in school as a child; patient 5 could not read the questions, and the interviewer read them to the patient and also filled out the worksheet with their answers; patient 11 did not want to fill out the worksheet alone but was happy to allow the interviewer to do so; patient 19 had Parkinsonism and, therefore, was unable to write on the worksheet; the interviewer filled out the worksheet for this patient.

### Challenge Completing the Worksheet Before Discharge

There were also logistical challenges with completing the 4Ms worksheet, including not having enough space on the worksheet to adequately answer the questions, as there are only so many lines available to write under each element. Limited time was another challenge, as shown in this example: Patient 17’s interview was performed just before they were discharged, so it felt rushed as the patient was getting ready to leave. The interviewer and patient were also interrupted twice during the interview.

Overall, the use of the 4Ms worksheet required additional personnel to help interpret questions and fill out question items. Any downtime was used to finish this sentence, which provided opportunity to complete the worksheet.

## DISCUSSION

We found that emergency clinicians have a good understanding of problem-oriented goals of care but not underlying goals. Further, the potential usability of 4Ms worksheet to facilitate the conversation between patients and emergency clinicians faces challenges. A successful implementation of the 4Ms framework in the ED is key in integrating emergency care into the age-friendly health system. Themes we identified highlight the unique aspect of 4Ms and the worksheet to facilitate such care.

In terms of understanding patient goals of care, we identified clinician/patient concordance, understanding underlying goals of care, and underlying goals of care discrepancy. In most interviews, patients and clinicians agreed on a problem-oriented goal related to the patient’s reason for presenting to the ED. However, we found that most emergency clinicians did not evoke the patient’s underlying goals for the visit. The literature on using the 4Ms framework to elicit goals of care among older adults in the ED is limited. One study found that when discussing “what matters” to the patient, emergency clinicians and patients agreed that discharging home or reduction/resolving of symptoms was a high priority, but emergency clinicians often did not identify the patients’ desire to return to prior functional ability.^18^ Our study findings are similar in that many patients interviewed had underlying goals, but these goals were not described by the clinician.

In terms of barriers in using the 4Ms worksheet, we identified worksheet-to-discussion discrepancy, challenges using the worksheet, and challenges completing the worksheet before discharge. Nearly half of the patients did not feel comfortable filling out the worksheet and required the interviewer to assist them in doing so. This was due to a variety of reasons. In instances in which the patients did fill out the worksheet, there was often a discrepancy between what was written on the worksheet and what was discussed during the interview. In one instance the usage of the worksheet and discussion felt rushed due to the patient’s impending discharge. In these cases, patients may not have fully described their goals, and interviewers may not have asked more in-depth questions about their goals.^10^

There is limited literature on the use of the 4Ms worksheet in the context of the ED. One paper suggested using a team approach to evaluating the 4Ms in elderly patients in the ED (eg, pharmacists should discuss medications, and social workers should discuss mobility). Another study used transitional care nurses in the ED to evaluate elderly patients for cognition and mobility and found that using such care nurses decreased admissions and readmissions to the hospital.^19^ ^,^ ^20^ Our study is unique in that we used a worksheet based on the 4Ms framework and evaluated its feasibility for use in the ED. Given the amount of time that the discussions take, we suggest using a team approach or have a dedicated person to have 4Ms discussions with patients.

### Strengths and Limitations

Strengths of our study include the in-depth discussion with patients, which allowed us to understand their goals and what matters to them. Another strength is that we had multiple members of the research team coding the same interviews; this allowed us to add more objective and diverse points of view. However, there were also several limitations to our study. The sample size was limited, and we were unable to recruit a sufficient number of APPs. Since we recruited these subjects during active clinical care, the time that physicians/APPs had for this interview was about 1-5 minutes, which may have caused bias. Patients were enrolled from a single center, limiting transferability to other health systems and geographic regions. The interviews were not recorded; so verbatim quotes were not possible in all cases, which may have caused recall bias. Also, we did not have access to demographic variables, again affecting transferability of findings. There was no quantitative measurement of discrepancy in the coding results.

### Future Implications

Implications of use of the 4Ms worksheet for clinicians include increased time spent with patients and greater patient satisfaction, but also includes increased probability of falling behind in patient care. Implications of use of the 4Ms worksheet for patients include increased safety and needs being met, potential avoidance of hospital admission, and improved patient outcomes. It would be interesting to see whether early use of the 4Ms worksheet in the ED course with subsequent availability for ED clinicians allows greater concordance in the goals of care. The use of the 4Ms framework for emergency care is not fully developed, and the use of the worksheet can facilitate situation-specific care (eg, discharge planning).

## CONCLUSION

We found that using the 4Ms framework as a guide for the care of older adult patients in the ED can help elicit underlying goals of care. We were able to answer whether patients’ goals of care were congruent with what the emergency clinician believed the patients’ goals were related to the presenting problem and with the patients’ underlying goals. Our study also found that the use of the 4Ms worksheet in the ED needs more research on how to best incorporate it into the care of older adult patients, as many older adults may need additional assistance to fill it out. We suggest that the 4Ms worksheet can be used with older patients who present to the ED to guide conversations with clinicians. This study is preliminary, and requires a validation study to further test the worksheet’s utility and acceptability in the ED.

## Supplementary Information




