# Supplementary material for: Usability of the 4Ms Worksheet in the Emergency Department for Older Patients: A Qualitative Study
Source: West J Emerg Med. 2024 Jan 25;25(2):230–6. doi: 10.5811/westjem.18088 (PMC11000547; doi:10.5811/westjem.18088)
Supplement: Supplementary file 1 [file wjem-25-230-s001.pdf]

Appendix 1.

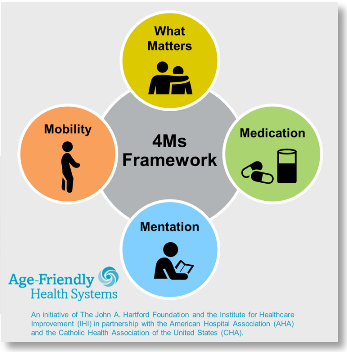

My Life • My Health • My Goals
My 4Ms

Make a difference in how your health care works for you by using the **4Ms of Age-Friendly Care:** What **M**atters, **M**edications, **M**entation, & **M**obility! The 4Ms are explained below – then flip the page and write your own 4Ms down on the other side. Put your 4Ms on your fridge, update them as needed, and take them with you to all your healthcare visits!

WHAT MATTERS

Receive the best care possible by telling your healthcare providers WHAT MATTERS to you. Think about what is most important to you in life; things you want your health care team to understand about you as an individual. Who are the most important people in your life? What do you view as essential to your quality of health and well-being?

If something were to happen to you, who would you turn to for help making healthcare decisions?

MEDICATION

Understanding your medications and what they do is important. Our bodies change with age in ways that can increase the chances of side effects from medications. One way to help prevent complications from medicine is to understand why you are taking them and to address any concerns about them with your healthcare provider.

MENTATION

Thinking and memory, mood, and mental health matter! Just like your body changes with age, so can your brain. Ways to support mentation include being engaged in meaningful activities and managing stress and anxiety. Trying new ways to relax and connect may be helpful.

For example:

- *Deep breathing*
- *Walking*
- *Doing things that bring you joy*
- *Taking breaks from the news*
- *Helping others by volunteering*
- *Paying attention to nature*
- *Connecting with family, friends, and neighbors*

MOBILITY

Stay as physically active as possible. Set a realistic daily mobility goal, something active you can do every day. This goal may start small and then change as you get more physically active.

For example:

- *Walk outside for 15 minutes every day*
- *Do daily chair exercises for 10 minutes first thing in the morning*
- *Light yoga or tai chi online or with a friend*

For more information about how to use this card, email **agefriendlycarepa@psu.edu**

PennState College of Nursing | Center of Geriatric Nursing Excellence

Age-Friendly Care, PA — A Collaborative for Age- and Dementia-Friendly Care

The Primary Health Network — Embracing Excellence in Healthcare

My Life • My Health • My Goals
My 4Ms

Health is a team effort, and YOU are in the driver's seat! Complete the 4Ms below and take this sheet with you to your next healthcare visit. Look at your answers before each visit and see if they change over time.

PREFERRED NAME: ______________________________

WHAT MATTERS

Take a moment and think about who you are and what you are facing right now, what is the most important thing for that comes to mind?

____

____

____

MEDICATION

Are there any medications that you feel unsure about why you are taking them, or how to take them? List your concerns and questions.

____

____

____

MIND

List 2 things you do that help you relax, & be positive. Let your provider know if you are having sadness or concerns about your memory or thinking.

____

____

____

MOBILITY

Set an achievable daily mobility goal for yourself.

____

____

The information on this flyer is not intended or implied to be a substitute for professional medical advice, diagnosis or treatment. All content, including text, graphics, images and information, contained on or available through this flyer is for general information purposes only. If you have any questions or concerns regarding your health, please reach out to your preferred primary care provider. If you are experiencing a medical emergency, please call 911 or go to your nearest emergency room.
This work was created by Age-Friendly Care, PA at the Penn State College of Nursing, and is supported by the Health Resources and Services Administration (HRSA) of the U.S. Department of Health and Human Services (HHS), through the Geriatric Workforce Enhancement Program (grant no. U1QHP33075). The contents are those of the author(s) and do not necessarily represent the official views of, nor an endorsement, by HRSA, HHS or the U.S. Government.

Appendix 2.

COREQ (COnsolidated criteria for REporting Qualitative research) Checklist

A checklist of items that should be included in reports of qualitative research. You must report the page number in your manuscript where you consider each of the items listed in this checklist. If you have not included this information, either revise your manuscript accordingly before submitting or note N/A.

Topic	Item No.	Guide Questions/Description	Reported on Page No.
**Domain 1: Research team and reflexivity**			
Personal characteristics			
Interviewer/facilitator	1	Which author/s conducted the interview or focus group?	7
Credentials	2	What were the researcher's credentials? E.g. PhD, MD	Title Page
Occupation	3	What was their occupation at the time of the study?	NA
Gender	4	Was the researcher male or female?	NA
Experience and training	5	What experience or training did the researcher have?	7
Relationship with participants			
Relationship established	6	Was a relationship established prior to study commencement?	7
Participant knowledge of the interviewer	7	What did the participants know about the researcher? e.g. personal goals, reasons for doing the research	7
Interviewer characteristics	8	What characteristics were reported about the inter viewer/facilitator? e.g. Bias, assumptions, reasons and interests in the research topic	NA
**Domain 2: Study design**			
Theoretical framework			
Methodological orientation and Theory	9	What methodological orientation was stated to underpin the study? e.g. grounded theory, discourse analysis, ethnography, phenomenology, content analysis	8,9
Participant selection			
Sampling	10	How were participants selected? e.g. purposive, convenience, consecutive, snowball	7
Method of approach	11	How were participants approached? e.g. face-to-face, telephone, mail, email	7,8
Sample size	12	How many participants were in the study?	9
Non-participation	13	How many people refused to participate or dropped out? Reasons?	NA
Setting			
Setting of data collection	14	Where was the data collected? e.g. home, clinic, workplace	7
Presence of non-participants	15	Was anyone else present besides the participants and researchers?	NA
Description of sample	16	What are the important characteristics of the sample? e.g. demographic data, date	9,10
Data collection			
Interview guide	17	Were questions, prompts, guides provided by the authors? Was it pilot tested?	7
Repeat interviews	18	Were repeat inter views carried out? If yes, how many?	NA
Audio/visual recording	19	Did the research use audio or visual recording to collect the data?	NA
Field notes	20	Were field notes made during and/or after the inter view or focus group?	7,8
Duration	21	What was the duration of the inter views or focus group?	8
Data saturation	22	Was data saturation discussed?	???
Transcripts returned	23	Were transcripts returned to participants for comment and/or	NA

Topic	Item No.	Guide Questions/Description	Reported on Page No.
		correction?	
**Domain 3: analysis and findings**			
Data analysis			
Number of data coders	24	How many data coders coded the data?	8,9
Description of the coding tree	25	Did authors provide a description of the coding tree?	9
Derivation of themes	26	Were themes identified in advance or derived from the data?	10,15
Software	27	What software, if applicable, was used to manage the data?	8
Participant checking	28	Did participants provide feedback on the findings?	NA
Reporting			
Quotations presented	29	Were participant quotations presented to illustrate the themes/findings? Was each quotation identified? e.g. participant number	12,13
Data and findings consistent	30	Was there consistency between the data presented and the findings?	18
Clarity of major themes	31	Were major themes clearly presented in the findings?	11
Clarity of minor themes	32	Is there a description of diverse cases or discussion of minor themes?	NA

Developed from: Tong A, Sainsbury P, Craig J. Consolidated criteria for reporting qualitative research (COREQ): a 32-item checklist for interviews and focus groups. *International Journal for Quality in Health Care*. 2007. Volume 19, Number 6: pp. 349 – 357

Once you have completed this checklist, please save a copy and upload it as part of your submission. DO NOT include this checklist as part of the main manuscript document. It must be uploaded as a separate file.

Appendix 3.

4Ms ED Needs Assessment Scripts

Aims:

1) Gain knowledge of the goals of care of patients in the ED age 60 or older using the 4Ms framework.
2) Assess the usability of the 4Ms worksheet the context of ED visit
3) Briefly obtain the ED provider's perspective on the patient's goals of care for the purpose of identifying discrepancies between the patient perspective and the provider perspective.

Patient interview script:

Introductory script: "*Today we would like to talk to you about your goals of care while you are here with us so we can better understand how to achieve your goals of care in the emergency department. We think it is helpful to break down your goals of care into four categories, so we can better understand different areas of your life that affect you as a whole person. To do this, we want you to fill out a worksheet today that separates your goals into the four categories. We will explain more about these categories as we go through the worksheet together. In general, we will ask you about what matters most in your life. We want to know about your medications and how they affect you. We will ask you about your mood and how you keep your mind active. Lastly, we want to know about how you move around and any challenges you experience with your mobility. After you finish filling out the worksheet, we also want to ask you about anything you feel was left out or anything else you want to share about your goals of care today.*

Questions

Note: will consider adding probing questions- such as "Could you elaborate?" if there is a short response or no response.

0) Introductory question- "Tell us how your visit went today". Ask for a few more details to make this more conversational.

1) Gain knowledge of the patient's goals using 4Ms – What Matters

Script:

"The first category of the worksheet is about what matters most in your life. Knowing these aspects of your life is important because we want to provide care in a way that helps you do what is important to you and does not interfere with what is important to you. As you fill out this section, think about what you are facing in life right now and what worries you. Consider what in your life makes you happy and is important for you to keep doing. What is the most important thing in your life that comes to mind?"

2) Gain knowledge of the patient's goals using 4Ms – Medication

Script:

"The second category of the worksheet is about your medications. Our bodies change as we age in a way that can make medication side effects more likely. Knowing what medications you are taking and how they affect you is important so we can address concerns and potential problems. We want you to take a moment to think about what, if any, side effects or challenges you experience with your medications. What questions do you have about what your medications are for or how to take them? In this section we would like you to list any concerns or questions that come to mind about your medications."

3) Gain knowledge of the patient's goals using 4Ms – Mentation

Script:

"The third category of the worksheet is about your mood and memory. How you are feeling can affect your memory and concerns you may have with your memory can affect your mood. Both your memory and mood can impact your overall health. So, it is important to know how you manage your mood and keep your mind active, so we can care for you as a whole person. In this section we would like you to list two things you do that help you to relax or to feel calm and be positive. Then we want you to write what you do to keep your mind active.

4) Gain knowledge of the patient's goals using 4Ms – Mobility

Script:

"The last category of the worksheet is about your mobility and how you feel moving around. Knowing how you like to be active is important, so we can help make sure you are able to do the tasks and activities that matter to you for as long as possible. Also, knowing what movements or activities you may struggle with lets us know how to help you get moving the way you want. Right now, we want you to take a moment to think about ways you are physically active every day. Consider what physical activities are important for you to maintain, or what activities you want to feel better doing. Then we want you to write down an achievable daily mobility goal that you set for yourself keeping in mind the physical activities that are most important to you.

5) Patient perspective on how well goals of care were elicited/addressed by ED provider and usability of worksheet.

Script:

"Now that you have finished the worksheet, we want to ask you a few more questions to pull everything together and to give you an opportunity to share any other comments you may have for us. How did you find the usability of the worksheet today? For example, what did you like about the worksheet? What was challenging about the worksheet?"

"What, if any, goals of care do you have today that you feel were not covered by the worksheet?"

"Considering everything we discussed about you as a person, what are you most worried about today that is bringing you to the ED?"

"How well do you feel your ED provider understood what your goals are and what is most important to you today?"

"Do you have any additional thoughts or ideas you want to share before we finish our interview?"

RA Instructions:

Please create a field note immediately following the patient interview. In this note, please include your overall impression and non-verbal aspects of the interview that stood out to you. For example, what was the environment like in which you conducted the interview? Busy? Loud? Was there anyone else in the room? Did that person speak or give non-verbal ques at any time? What was your overall impression of the patient? Distracted? Engaged? What non-verbal ques did the patient provide and when? What stood out to you about this encounter?

Provider interview script:

Introductory script: "*Today we would like to interview you about aspects related to caring for adults 60 or older in the emergency department with the goal of performing a needs assessment for age-friendly methods of eliciting goals of care. We want to gain insight into how current practice patterns influence the perception of goals of care conversations between patients and providers.*

Questions

Note: will consider adding probing questions- such as "Could you elaborate?" if there is a short response or no response.

1) Provider perspective on patient's goals of care

Script:

“In your opinion, what are the main factors and concerns that bring the patient to the ED today?”

“Following the last question, what do you think matters most to the patient regarding their care today?”

“What additional comments do you have about the patient’s goals of care or your experience eliciting their goals of care today?”

Appendix 4.

Aim 1. Our objective was to gain knowledge of the goals of care of patients in the ED age 65 or older using the 4Ms framework. We conducted qualitative research using the semi-structured interviews from older ED patients and their caregivers.

Aim 2. We employed semi-structured interviews to evaluate the potential usability of the 4Ms worksheet to facilitate the conversation between patients, caregivers, and ED clinicians and whether ED clinicians have a good understanding of goals of care including both problem-oriented goals and deeper goals related to the 4Ms.

4Ms codebook study ID 1-20 (04/28/2022)

Theme/Code	Definitions	Quotes	Aims
**What matters**			
Family	Spending time with family is what matters. Being around his kids, wife, and other family members is the most important.<br><br>Getting together with family<br><br>Cooking for family<br><br>Including church, connections, social with family<br><br>Be around friends	On further probing: Spending time with family, helping her mom with cooking Christmas dinner to feed their large family (20+ grandkids), going to Church with her family, and spending time with her many grandchildren as well as doing things for them like making dresses or helping with their events.<br><br>Being with family is most important –pt 4<br><br>Being with family is most important (9)<br><br>Family seemed really important to patient 11 and also the source of her recent worsening of depression<br><br>“What makes them special” - Patient 19<br><br>Enjoy quality time with family (16)<br><br>He says	Aim 1

		spending time with his grandchildren is the most<br>important thing and he wants to be able "live long<br>enough" so that he can give his grandchildren "fond<br>memories of him." 17<br><br>Faith is important- 15<br><br>From notes- patient says he would like to be around his friends	
Home	Preference being in home rather than going to nursing home or staying in the hospital<br><br>Patient owns a property	Patient was put in a (residential) home 2 years ago but able to leave that place 1 year ago and live by self<br><br>Patient likes to spend time at home and would like to continue to do so (9)<br><br>Patient is a retired farmer and would like to be able to stay healthy enough to care for his large property<br><br>His ability to be physically active and care for his property is really important to him and his goal is to keep himself well enough to perform these activities given his heart history	Aim 1
Diagnosis/Screening	Doing a diagnostic test to find out if one has malignancy<br><br>Learning about diagnosis<br><br>Including screening	Patient is concerned that she had an MI and hopes that is not the case<br><br>Something spotted on PET scan, would like to know what they find	Aim 1

		R/O risk of paralysis as he fell and had arm weakness and numbness (17)	
Cooking	Be able to cook for independence<br><br>Cooking for fun	patient would like to “be able to cook for myself”<br><br>Patient 10 also enjoys cooking	Aim 1
Coordinated care	Patient or provider driven collaboration between hospital teams	Patient drove from 1.5 hours away to receive care at this hospital because these doctors know her and know her complex medical history<br><br>Also patient 17 everyone does their job right- things are going in correct order (incomplete here)<br><br>Healthcare workers do their job correctly (17)	Aim 1
Living longer	Perspective on the limited life due to illness.	“I want to live as long as I can” in the setting of recent cancer diagnosis<br><br>Two years ago he was told he had 6 months to live and so they sent him to a "home" to live for the rest of his life. He was able to leave the "home" a year ago and live by himself, which he would like to continue to do. However, he is unsure if this will happen. Going forward he would like to be around friends, be able to cook for himself, outlive the people who told him he would die 2 years ago, and continue with his current state of	Aim 1

		mobility w/walker and wheelchair.<br><br>Patient said he would like to “outlive the people that told him he would die 2 years ago”<br><br>(Record_id 12) Being able to "stay in shape" and for him to be able to "live longer."<br><br>"didn't want to die” pt 14	
Problem oriented expectation	Most ED visits are about problem oriented visit- so, that may make it easy to set an expectation.	At today's visit what matters most to her is that she gets her gallbladder out so she isn't sick anymore.<br><br>Figure out if clot is present or not – patient 13<br><br>Actively listens to her concern and responds to them. “the healthcare team listens to her and her concerns.” Patient 18	Aim 1
Getting better	How best to care for self<br>Symptom relief<br><br>Getting procedure done so one does not get sick anymore	Getting information about …what I can do to get better (03)<br>"get better and to stay active" (08)<br><br>Mike came in to be evaluated for 1 week of worsening fatigue. He runs a relator business were he has to be very physically active and his fatigue was interfering with his ability to do his job. His wife prompted him to take his pulse and found that it was slow, so she	Aim1

		encouraged him to come to the ED today. (10)<br><br>From notes- patient is to get her gallbladder removed because she has been sick and would like this done because she “never gets sick.”<br><br>Pain relief from tumor burden (16)<br><br>Constipation, abdominal pain, Patient has a significant heart history and is here today for palpitations.	
Obtaining healthcare information	Need to access information on diagnosis and prevention	Getting information about my own healthcare (03)<br><br>“Understanding what is happening to me” (20)	Aim 1
Independence	Care that promotes or maintains patient independence<br>independent as long as possible, being independent despite medical problems	Patient lives independently and recently began suffering falls – she adamantly wants to avoid needing to live in a facility after a recent bad experience in a facility following another fall. -Patient 3	Aim 1
Patient education	Patient or provider initiated patient education about medical condition, treatment, or prevention	Patient wanted information to better care for herself to avoid falls in the future - patient 3<br><br>Patient 18 – “She wants to be more careful and avoid falls in the future.”	Aim 1
Transportation	To have means to transport to ED and home, such as car ride, help from family or friend.	At the time of the interview, Sue said her biggest concern today was how she would get home. …Now her major	Aim 1

	Patient reports what matters as being physically able to keep working	concern is how she will get home because she left her phone at home, she has many mobility issues, and does not have help from family currently. (11)<br><br>Came in with dizziness and needs to be able to ride bike for transportation – patient 12<br><br>Needs to maintain physical strength to be able to work – Patient 12	
Active hobby	Any type of hobby that matter to patient<br>Football- being able to watch football	Patient 10 reported watching football matters to him and helps him relieve stress	Aim 1
Stay active	Being able to do enjoyable physical activities<br><br>How best to care for self<br>Symptom relief<br><br>Getting procedure done so one does not sick anymore	"get better and to stay active" (08)<br><br>Patient 15 (per notes)- being active and playing golf. Doesn’t want to be “slowed down” by illness<br><br>Being active with several hobbies (kayaking, hiking, racquetball, walking dog) – patient 13<br><br>He was most worried that whatever was causing his pain would "slow him<br>down" again like his prostate cancer treatment as<br>being active is extremely important to him. P15	Aim 1

Sleep	if he had to stay at the hospital he wants the healthcare team to know that he would need a CPAP and he would like it to be dark so he is able to sleep. As soon as he is able to go home he would like to sleep.<br><br>If need to stay in the hospital, it has to be comfortable.	Patient said he would prefer to go home than stay at the hospital, but if needed to stay at hospital he would need a CPAP and dark room in order to be able to sleep	Aim 1
Health/Live longer	Stay healthy and live longer	Per patient (14)- has chest pain and “didn’t want to die.” Wants Health and living longer matter to him most<br><br>“staying alive and staying healthy” (17)	Aim 1
Doing good for others	Doing good for others	Patient 15 (per notes)- it is important for him to do good for others<br><br>Patient 18 – “To put a smile on others.”<br><br>Patient 19 – “She wants to be able to help people if she can”	Aim 1
Listening	Important that healthcare team listens to concerns	Patient 18 – “she said that the healthcare team listens to her and her concerns. That they listen to her when she has pain, is thirsty, or hungry”	Aim 1
Proper care received	Receiving appropriate level of care	Everything was done in the correct order and everyone did their job.	Aim 1

		He felt that the healthcare team here was very professional, nice, humorous, and kept him "out of being depressed." When asked if the ED provider addressed his concerns and goals of care he said<br>"yeah, everyone was nice."17<br><br>He was upset because he was transported between three different hospitals and he wasn't able to eat anything in case he needed a procedure. 17	
**Medication**			
Discordant recommendation	One physician recommends to take medication, others not to.	"There is some confusion about the senna she takes because some doctors say she is fine to use it often (GI docs) and others say not to use it so much.<br>" Patient 20	Aim 1
Compliance	Daily medication compliance, daily routine to take them as prescribed	Sometimes I get so busy I forget to take them [medications]. When I realize, [I think] tomorrow is a new day and I try to do better each day.<br><br>He doesn't like to take his medications, but he knows that it's "necessary 17	Aim 1
Polypharmacy	Too many medications,	He is prescribed a lot of meds and he doesn't know why/what they are for<br><br>He was off a lot of his medications when he fell, which is the reason that brought him to the hospital.	Aim 1

		Patient 14- on many meds (10-12) and unsure of names, what they are for, and when to take them<br><br>He did said he was on 10 or 12 medications and is unsure the names of<br>most of them or what they are for or when to take<br>them. P14<br><br>He would like to be on as little meds as possible.	
Side effects	Unexpected or undesirable effect due to medication<br><br>Medication complications and/or identification of medication problems in the ED	Drowsiness and bleeding easily from anticoagulants<br><br>Bleeding issues related to anticoagulant prompting medication change – patient 4<br><br>Lisinopril (BP medication) as a potential offending agent for dizziness/syncope-patient 12<br>Patient has had several issues with chemotherapy complications and came to this ED concerned for another complication. Also admits to feeling “foggy” with meds prior to chemo, which has been worse with addition of chemo. Also admits to worsening fatigue since chemo that is interfering with activities of enjoyment and resulting in weakness walking	Aim 1

		Patient 20 – "She also mentions that she takes duloxetine and worries about it causing worsening constipation."	
Patient knowledge	Patient doesn't know what meds one is getting, not only what but why, and dosing issues.	From notes- patient is referring specifically to the meds she is receiving at the hospital. She says it is all "blurry." She doesn't know what she is getting or why<br><br>She reports not being sure what each medication is for but states she is not concerned about this as she trusts her doctors "wouldn't prescribe something [she] doesn't need" (03)<br><br>He did say he was on 10 or 12 medications and is unsure the names of<br>most of them or what they are for or when to take them. P14<br><br>Patient reports difficulty figuring out how much insulin to give himself – patient 4<br><br>Patient 19 – "When asked about knowing her medications and why she takes them she replied, "yes and no." She says she "should know what I take and why I take it.""<br><br>Unsure what all meds he is taking/what they	Aim 2 – not a patient goal so not aim 1, but worksheet did identify gap in patient education

		are for, but does “what the doctor says” (17)	
Pill box	Patient uses a pill box to organize meds	“Only takes a few medications and organizes them in a pill box” – patient 10. I think this is a repeat code as other patients have used pill boxes<br><br>Patient 11 also uses a pill box<br><br>Patient 15 uses a pill box and therefore denies trouble with meds<br><br>Pill box use therefore no trouble with meds (16)<br><br>Pills organized in a “medicine caddy” (17)	Aim 1<br>Aim 2
Blind trust	Trusting provider/caregiver because of difficulty understanding medications	She is trusting the healthcare team to provide her with the appropriate meds.<br><br>Her husband takes care of her medications. Patient 19.<br><br>He is not sure of all the medications that he takes as his wife is in charge of that.<br><br>Does "what the doctor says." 17	Aim 1
Help with managing meds	Patient's wife is a retired nurse and helps him with his medications. He is not sure of all the medications that he takes as his wife is in charge of that.	Wife is a former nurse and manages his meds for him<br>Wife handles medications and she has no concerns/feels they are all necessary<br><br>caregiver that organizes	Aim 1

		his pills into a "medicine caddy" that holds 4 weeks worth of medicines. He said that sometimes he will ask his caregiver if he can organize them as it gives him something to do. He usually will pick up his meds, but if he can't, his caregiver will. 17	
**Mobility**			
Strength	Get strong and get on feet again	Patient 11 also expressed trouble with strength in terms of carrying her oxygen<br><br>Patient 20 – "She also has a goal to do strength training such as weights or resistance bands."	Aim 1
Active	Patient would like to stay active in terms of physical activity	From notes- Patient is very active and would like to stay active so she can continue to live independently<br><br>Patient would like to stay active to take care of his large property – "Just be active"(8)<br><br>Patient 15- stay active and play golf<br><br>Patient 13 usually has very good mobility and enjoys activities that require a lot of physical activity but is recovering from knee surgery so hasn't been as active<br><br>Patient 18 – "To stay active, Ladonna says she likes to ride her bike, jog, do leg lifts in	Aim 1

		the morning, and do "twists."<br><br>"I walk the hall daily, plan to do double that. Try to be more active at home and not so much sitting." (03)<br><br>Husband tells it is important for her to continue to exercise, walk, and resume yoga. Patient 19.<br><br>Patient 20 – "goal to walk at least 30 minutes a day."	
Functional mobility	Physically active to accomplish task	"Move to do projects around the house" (04)<br><br>Job involves working with hands and has arthritis limiting his ability to work patient 12<br><br>From notes- patient mows her own lawn and would like to continue to do so<br><br>Mike's job responsibilities require him to be very physically active doing walkthroughs of properties, putting up signs, and driving around Iowa City.(10)<br><br>"To stay independent and take care of herself, dog, and cat" Patient 18<br><br>Patient would like mobility without pain (16)	Aim 1

Transportation	Driving or getting a ride to access	If can't drive, need to call for ride to leave home. can't get into vans due to mobility.<br><br>Patient cannot drive and cannot get into a van by herself due to limited mobility. Her only transportation is to call for a ride and it must be a small car. patient 3<br><br>Patient 11 has transportation limitations due to her mobility and car situation	Aim 1
Independence	Being able to move to do activity of daily living with or without assisted device.	"To stay independent and take care of herself, dog, and cat" Patient 18<br><br>Has a walker and wheelchair to get around and would like to have the same mobility as he had before current admission<br><br>He is able to slowly walk around the house on his own.<br><br>Patient 18 – "goals for mobility are to remain independent"	Aim 1
Rehab	Rehab for improved physical activity<br><br>Physical therapy	Patient enjoys going to cardiac rehab 2-3 times a week after previous MIs. He feels he is getting a lot out of it and it will help him stay active<br><br>After this admission he would like to still have the same mobility that he had before. He would also like to work with PT so that this can happen.	Aim 1

		Patient reports he will need to go to physical therapy to improve neck mobility (17)	
Limitations	Pain as a health or physical factor to limit mobility	Arthritis in hand (thumbs) slow him down as he assembles and fixes bike. (Case 12)<br><br>Sue reports many mobility issues. She needs oxygen to go out and has difficulty carrying the oxygen with her. She has knee pain, diabetic neuropathy, and an unsteady gait so she needs a motorized chair if she goes anywhere. Because of this she says she rarely goes out and only goes to 4 stores that have chairs for her. She would like to be able to get out of the house more often, but feels limited by her pain, strength, and oxygen needs. (11)<br><br>Patient has arthritis which allows limited mobility<br><br>“One thing that she wishes she could do is run, but she is unable to.” Patient 20<br><br>He reports some trouble with walking in the winter<br>due to more frequent flares of his sciatic nerve	Aim 1

		pain. He walks a lot so this pain and difficulty walking bothers him often in the winter months but<br>less in the summer. P14<br><br>Walking is painful from prior surgeries and tumor compressing spine (16)<br><br>Patient cannot do much physical activity due to severe back pain, heart problems, and SOB (17)<br><br>Patient reports difficulty walking due to PVD and wants to be able to walk more – pt 4<br><br>Patient 11 has difficulty walking due to pain, diabetic neuropathy, and burden of carrying oxygen<br><br>Patient 14 walks often but has trouble with sciatic nerve pain, especially in winter months	
Inactivity	Choosing to be inactive	He considers himself a "professional couch potato." (09)<br><br>When asked about physical activity, he laughed and<br>said, he can't do it.<br>17	Aim 1
**Mentation**			
Memory	Trouble remembering names with faces, trouble recalling names, forgetting walker, recalling events	She is not worried about losing her memory.<br><br>He is able to have a conversation and has good long-term memory, but his short-	Aim 1

		term memory is not very good<br><br>She often repeats words/phrases and forgets things. Patient 18	
Depressed mood	Identification of depressed mood	Patient endorses weekly feelings of depression related to adjusting to cancer diagnosis<br><br>*Patient reports feelings of depression related to chronic pain and losing self-sufficiency -patient 4<br><br>Sue states she has been feeling increasingly depressed lately. She notes that she recently became somewhat estranged from her daughter and this is really impacting her emotionally. She doesn't exactly know why her daughter stopped talking to her, but she is very upset about it. She reports difficulty sleeping and frequent distressing thoughts about the situation.(11)<br><br>Drinks Whiskey to escape thoughts of depression. Case 12<br><br>Did endorse feeling upset at times. He likes to take a<br>hot shower to relax. He also smokes cigarettes to<br>relax.<br><br>Depressed mood in thinking about age and	Aim 1

		not being able to do active things like “roller skating or bowling” (17)	
Anger	He endorses feelings of anger when he cannot be as self-sufficient anymore.<br><br>Also includes frustration	Patient reports anger related to his pain and losing self-sufficiency – patient 4<br><br>Patient 11 reports frustration about her family situation and not understanding why her daughter won’t talk to her	Aim 1
Stress	Patient reports experiencing stress<br><br>A way to cope with stress<br><br>Worries about diagnosis	Patient 10 reports stress related to work<br><br>She is currently reading a sci-fi book and also likes to escape into books for stress relief-case 13<br><br>Reading and hidden object games to escape from stress of cancer (16)<br><br>New cancer diagnosis causes worry (16)	Aim 1
Positive Mood	Patient gets a lot of enjoyment from being active outdoors, spending time with family, and watching sports and these activities help maintain a positive mood.<br><br>Include work to stay active in mind<br><br>He says that he is generally in a good mood, but has a better mood when he is at home.	Spending time with family, spending time outdoors, and watching sports keeps a positive mood<br><br>Stays positive by doing activities he enjoys—movies, music, cooking (17)<br><br>“TV and exercises, grand kids, large yard, and fishing” (08)<br><br>Patient states he is usually in a good mood, but his mood is better at home. He states “there is no place like home” (9)	Aim 1

		Uses keeping busy at work to distract from depression – patient 12<br><br>Patient 18 – "has a positive attitude. She considers herself a "pretty understanding person.""<br><br>"there is no place like home." (09)<br><br>Patient states she always "has a positive attitude."<br><br>"Not a quitter" patient 18<br><br>Healthcare team helped him keep a positive attitude by keeping him "out of being depressed." He says they were professional, nice, funny (17)	
Dementia	Cognitive impairment	Patient has dementia according to wife- main problem at this point is his short-term memory but he is able to hold a good conversation and his long-term memory is good<br><br>Patient had family member with Alzheimer's and does not want this disease<br><br>He feels that he forgets easier than he used to, but he doesn't really do anything to relax, keep positive, or keep his mind active. He just doesn't want Alzheimer's like one of	Aim 1

		his family members had.<br>He does sometimes worry about dementia but its "not that serious" right now. 17<br><br>She says her "brain doesn't want to wake up till noon." She says this happens constantly. She does worry about getting dementia or Alzheimers. She mentions that she used to work in research but recently began a new job within the last year because she is unable to multitask now. Patient 20	
Relaxing	Engage in a hobby to relax<br>Hot showers to relax<br>Reads to relax<br>Plays golf to relax<br>Watches sports to relax	He plays golf, reads, and watches sports to relax P15<br>Patient 14 (per notes)- patient likes to take a hot shower to relax<br>Patient 15 (per notes)- reads to relax<br>Patient 15 (per notes)- plays golf to relax<br>Patient 13 uses reading to cope with stress and keep mind active<br>Patient 19 – “editing a manuscript of letter she sent when she went to Africa. She is also editing a manuscript of a cookbook.”<br>Patient 15 (per notes)- watches sports to relax	Aim 1
Being inactive	Protentional contributor for depression	"being laid up in bed is the worst for him." wife 15	Aim 1

**Others (Aim 2-3)**			
Provider/Patient Concordance	Problem oriented goals of care being concordant.	patient thinks her concerns/goals have been addressed well by staff<br><br>*Both patient and provider arrived at goal of care is evaluation after fall and to be able to go home to continue living independently – patient3<br><br>*Both patient and provider arrived at goal of care of addressing symptoms of constipation and abdominal pain -patient 4<br>Both patient and provider agree the main concern bringing the patient in today is chest pain (9)<br><br>Both patient and provider agreed addressing symptom of fatigue was the goal of care – patient 10<br><br>Both patient and provider agreed that ruling out serious cardiac pathology was the main goal of care – patient 11<br><br>Patient 20 – " Patient is in the ED because of syncope or seizure and the goal is to determine the etiology of her symptoms."	Aim 2/3
Patient provider concordance	Patient and provider arrive at concordant	Both patient and provider identified getting the patient's	Aim 3

	perceptions of the goals of care	heart rate under control as a main goal<br><br>Both the patient and provider arrived at the main concern of addressing the fever today and coordinating care to address recurrent fevers related to chemo<br><br>Both the patient and the provider identified fatigue as the patients primary reason for seeking care today. The patient expressed that he wants his fatigue symptoms resolved so he can get back to work and enjoying cooking and football. The providers goal of care was to get the patient admitted for a pacemaker. (10)<br><br>(provider/patient concordance) Both the patient and provider identified a fear that the patient's chest pain may point to serious cardiac pathology given the patient's history. The provider elicited the foreboding feeling the patient was having as well.(11)<br><br>Both patient and provider understand that the patient's chest pain is what matters most (14)<br><br>Both patient and provider wanted to address abdominal pain (15)	

		Both patient and provider agreed pain management is main goal of care (16)<br><br>Patient felt he was treated well and when asked if the provider addressed his concerns he said “yeah, everyone was nice,” however there was no provider perspective to compare to (17)	
Worksheet to discussion discrepancy	Patient answer in the worksheet does not reflect answer in discussion	Patient answered no to medication problems but discussed many issues related to medications<br><br>*Patient discussed information relevant to discharge/disposition and revealed opportunity for education about medications not captured in her worksheet answers. - pt 3<br><br>*Patient 4 had issues with this as well	Aim 2
Unease	Patient feeling unease with the worksheet or 4Ms	Patient began fidgeting with the sheets and expressed discomfort with filling out worksheets stating that unease with worksheets extended back to being in school as a child<br><br>Sue did not want to fill out the worksheet herself but was very happy to talk with me. I offered to fill it out for	Aim 2

		her and she preferred that option.	
Barriers to using worksheet	Ability to read and understand the 4Ms form	Wasn't able to read the questions so I, the interviewer, had to read them to him. Was somewhat confusing, but able to complete the questions.	Aim 2
Understanding underlying goals of care	There seemed to be a slight discrepancy between the patient and provider regarding the underlying goal of care, which seemed to be maintaining the patient's independence.<br><br>Patient and provider both identified getting the patient's heart rate under control as a goal. It was not clear that the provider understood the underlying concern of that patient, which was that his heart conditions might inhibit his ability to be physically active the way he is used to being.	Unclear whether physician understood the underlying reason for the patient to get control of his heart rate—to be physically active<br><br>Unclear if patient had an underlying goal to stay in a warm bed at the hospital (patient is homeless), but ED physician mentioned this might be the case (14)<br><br>They differed on the underlying goal of care which<br>take note) to the patient was making sure he could maintain<br>his active lifestyle with whatever was causing his<br>pain. To the provider what mattered most to the<br>patient was treating cholecystitis. P15	Aim 2/3
Worksheet not preferred	Patient preferred to discuss instead of filling out the worksheet	Patient 10 wanted to discuss and preferred someone else fill out the worksheet<br>Patient 11 as well<br>Patient 12 preferred that interviewer fills out worksheet.	Aim 2

		Patient 14, 15 did not prefer either.<br><br>He did not want to write on<br>the worksheet and preferred I fill it out while we<br>talked. P14<br><br>He did not want to fill out the worksheet and Open ended question. preferred that I fill it out as we talked. P15<br><br>Patient preferred interviewer to fill out worksheet during discussion (16) Also (17)	
Underlying goals of care discrepancy	Patient and provider do not arrive at the same underlying goals of care.<br><br>Interpretation of what matters for patient and ED provider is often discrepant	The provider mentioned that the main goal of care was pain relief and that the patient would rather be at home and "soil himself" then be at the VA<br>hospital. (Patient wants to work on PT and gain strength, get off some medications)<br><br>Patient 4 – provider identified improving symptoms as the main goal of care but the patient identified improving independence as a main goal	Aim 3
Worksheet included all that matters	Worksheet addressed all concerns and what matters to the patient and their care	Patient 14 (per notes)- he did not feel the worksheet left anything out in terms of what matters to him and his care	Aim 2
Indeterminate concordance/discordance	Unable to determine concordance versus	Patient 18 – "Currently the provider is unsure	Aim 2

	discordance due to timing or type of response	what is going on with the patient. He has not had a discussion about her goals with her yet”<br><br>Patient 19 – “When asked about eliciting the patient's goals of care, he replied that the patient was handed off to him and so he did not have that discussion with the patient.”	
Challenge completing worksheet before DC?		Interview was performed just before the patient was discharged, so it felt a little rushed. We were interrupted twice during the interview and<br>I had to leave the room the first time and come back. He was wearing a c-collar during the interview. He was happy to chat with me, but was also excited to be discharged. He also had a call to make so that contributed to the interview<br>feeling rushed as well<br>17	
